# News Media Use and Crime Perceptions: The Dual Role of Ideology

**DOI:** 10.1080/15205436.2025.2471866

**Published:** 2025-03-07

**Authors:** Dennis Andersson

**Affiliations:** Department of Journalism, Media and Communication, Goteborgs Universitet, Gothenburg, Sweden

## Abstract

This study employs a three-wave panel survey in Sweden to test the Differential Susceptibility to Media Effects Model (DSMM) regarding crime perceptions. It assesses DSMM using political ideology as a susceptibility variable, revealing ideology’s role as a predictor of news media use and in shaping citizens’ perceptions. This paper investigates how this process depends on socio-economic and socio-cultural dimensions. Results lend partial support for assumptions made by the DSMM, indicating that individuals rely on their ideological predispositions in news selection. This work highlights ideology’s role in selective exposure and its role in evolving crime perceptions, emphasizing the importance of individualized modeling in understanding media effects in an expanding media landscape.

Violent crime has become a major public issue in Sweden. According to the results of the annual Society, Opinion and Media (SOM) survey,^1^^1^The SOM Institute has conducted studies centered around society, public opinion, and media habits amongst the Swedish public since 1986. It is a university-based research organization and provides a national infrastructure for survey data. this issue, which had been the tenth most important problem in 2015, became the most important one among the Swedish public in 2021 (Martinsson & Andersson, 2022). Violent crime is also a salient issue on the media and public agenda, receiving both national and international attention (e.g. Raagaard, 2021; Skogelin, 2021).

Media effects studies, often rooted in cultivation theory, explore citizens’ perceptions of crime development (e.g. Davis et al., 1999; Gerbner & Gross, 1976; O’Connell & Whelan, 1996; Pfeiffer et al., 2005; Shi et al., 2019). Negative views and perceptions of increasing crime, irrespective of current rates, are associated with media exposure (Davis et al., 1999; O’Connell & Whelan, 1996; Pfeiffer et al., 2005). However, the increasing number of outlets within the contemporary media environment has turned media use into a less collective and more individualized matter (Valkenburg & Oliver, 2019), motivating citizens to select messages matching their own beliefs and attitudes (Stroud, 2017).

The Differential Susceptibility to Media Effects Model (DSMM) accounts for these changes by exploring the importance of differential susceptibility variables in selectivity and responsiveness to media use—i.e. media effects—a combination of an individual’s dispositional features such as predisposed beliefs, situational conditions like societal circumstances or events, and an individual’s social context, where certain norms and behavior are favored (Valkenburg & Oliver, 2019). Susceptibility variables play a dual role, predicting media engagement and moderating media effects on outcomes like attitudes or societal perceptions (Valkenburg & Peter, 2013).

This study explores whether political ideology, a predictor of selective exposure (Stroud, 2010), conditions news media effects on societal perception trends of violent crime, testing the assumptions of the DSMM (Valkenburg & Oliver, 2019). This enhances our understanding of the relationship between ideology, news use, and societal perception in a fragmented media environment.

Traditionally, the political cleavages in Europe and in the United States have been situated around the economic conflict between labor and capital, known as the political left-right dimension (Inglehart, 2017; Jost, 2017). Yet, an alternative cultural dimension focusing on post-materialistic values has gained prominence (e.g. Hooghe et al., 2002; Inglehart, 1971; Kitschelt, 1994). This ideological dimension is receiving growing attention (Hooghe et al., 2002), and may be a crucial susceptibility variable for news consumption affecting perceptions of violent crime development (Hooghe et al., 2002; Shi et al., 2019).

This paper contributes by testing DSMM assumptions using a three-wave panel survey to explore the dual role of ideology in predicting news use and conditioning media effects, examining perceptions of violent crime. The analysis delves into the dependency of this process on two ideological dimensions—socio-economic and socio-cultural.

## Media effects and perceptions of violent crime

This study examines citizens’ perceptions of the severity and directional trajectory of violent crime development in society. Such positive or negative societal perceptions are often closely tied to ideological predispositions (Jost et al., 2008). As societal or political issues become salient, they also become important to citizens (McCombs, 2014).

Many prior research have indicated that frequent media use tends to shape perceptions reflecting negative depictions of crime presented by the media (Gerbner & Gross, 1976; Gerbner et al., 1980; Velásquez et al., 2018). Cultivation theory focuses on television viewing patterns and the impact of messages on public perceptions related to violent crime (see Gerbner, 1998; Gerbner & Gross, 1976). Although contemporary empirical research observes similar patterns, the results remain somewhat ambiguous.

Focusing on newspaper news exposure frequency, O’Connell and Whelan (1996) link negative perceptions of crime rates to news consumption, finding perceptions independent of measured rates. Velásquez et al. (2018) similarly note spikes in negative crime news heightening negative crime perceptions, with a thrice-larger effect than positive news. Conversely, Chadee and Ditton (2005) find no link between news media consumption and crime perceptions. Shi et al. (2019) attribute this to cross-sectional data limitations, cautioning against interpreting media effects on dynamic processes like forming crime perceptions. They suggest that selective consumption of liberal (e.g., MSNBC) or conservative media (e.g., Fox News) shapes different perceptions of crime.

## The differential susceptibility to media effects model—the dual role of ideology

As the modern communication environment entails a tremendous amount of online and offline sources (Bennett & Iyengar, 2008; Prior, 2007), people are forced to become selective in their news consumption (Stroud, 2017). When exposed to massive flows of information, people may use their personal preferences to select what type of news or media they consume (Stroud, 2017). Valkenburg and Oliver (2019) argue that this tendency must lead researchers to focus less on collective mass communication and more on self-communication, as media use is becoming progressively individualized (Valkenburg et al., 2016).

Within the field of communication, media effects have been found to be small or moderate at best, and it is suggested that this is due to a suboptimal conceptualization of the media effect process. As the media environment expands, the relations between media variables such as media use, and non-media ones, such as individual differences like beliefs or values, become more important when attempting to explain both media use and the emergence of media effects (Valkenburg & Peter, 2013; Valkenburg et al., 2016). To remedy this, Valkenburg and Peter (2013) propose a more individualized model, the DSMM, founding its inferences on observations of the individual media user and exploiting individual variables.

**Figure 1.**
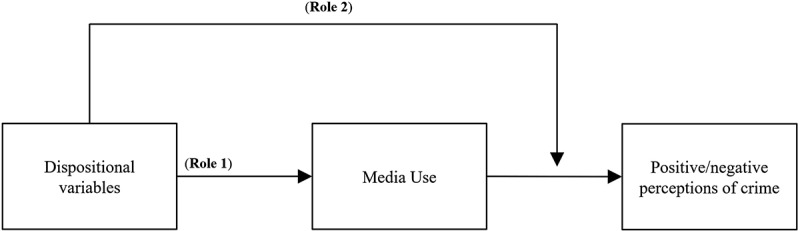
Theoretical model of the mediating moderating argument.

The DSMM builds on the assumption that media use has different effects on different people. Valkenburg and Peter (2013) state that media choices, media use and the subsequent media effects are dependent on differential susceptibility factors. According to the DSMM, media effects are generated through selectivity and responsiveness to media use, and dependent on differential susceptibility variables described as: an individual’s dispositional features such as predisposed beliefs, values, or attitudes, situational conditions such as societal circumstances or events, or one’s social context, where certain norms and behavior are favored (Klapper, 1960; Valkenburg & Oliver, 2019). Therefore, susceptibility should play multiple roles in conditioning media effects, where it first predicts news media use (role 1), and, second, stimulates the media effect by influencing people’s interpretation of the information in role 2 (Valkenburg & Peter, 2013). A visual depiction of this argument is provided in Figure 1.

The model depicts the argument of the Differential Susceptibility to Media Effects Model, that dispositional variables both predict media use, represented by role 1, and moderate the mediating effect of media use, illustrated by role 2 (Valkenburg & Peter, 2013).

Accordingly, this model should help in obtaining a better comprehension of how media effects emerge in an expanding and more individualized media environment. Thus, to understand media effects on perceptions of violent crime, we need to understand the roles of media and non-media variables, as well as the relationships between them (Valkenburg & Peter, 2013).

In criminology, susceptibility factors to media effects are explored through the “differential reception thesis” (Roche et al., 2016). This incorporates four hypotheses: (1) the substitution hypothesis, suggesting media strongly affects those with no crime experience, (2) the resonance hypothesis predicting the opposite, (3) affinity hypothesis expecting greater media effects in those resembling crime victims, and (4) the vulnerability hypothesis assuming audiences viewing themselves as crime-vulnerable are more receptive (Roche et al., 2016). However, the results have been inconsistent on which factors do produce strong media effects (Pickett et al., 2015; Roche et al., 2016). This study diverges from the emphasis on crime experience, opting for ideology as a dispositional predictor, aligning with prior media effect research on selective exposure (e.g., Knobloch-Westerwick, 2015; Stroud, 2017; Taber & Lodge, 2006). Yet, earlier studies may have overlooked shifts in the ideological landscape and somewhat neglected emerging political cleavages (Hooghe et al., 2002; Oscarsson et al., 2021).

The left-right ideological dimension, a dominant force in Western democracies for over a century, structures various aspects of political landscapes, including party systems, policy issues, voter behavior, and public opinion (e.g., Holmberg & Oscarsson, 2015; Oscarsson, 2020), as well as predicting ideologically congruent news exposure (e.g., Dahlgren et al., 2019; Shehata et al., 2022). While limited research has directly explored the link between political ideology, news consumption, and perceptions of violent crime, related studies highlight differences amongst ideological groups. Left-wing individuals tend to exhibit lower fear of criminal acts and more lenient punitive attitudes, whereas right-wing individuals often demonstrate greater fear of crime and a preference for harsher punishment (Gerber & Jackson, 2016).

However, politics have become increasingly polarized along a socio-cultural dimension (Oscarsson et al., 2021), driven by cultural value changes and high levels of existential security in the Western hemisphere (Inglehart, 1971, 2017). Long-term value changes and the rise of authoritarian populism have led elites and citizens to polarize along post-materialistic values, including criminality, environmental issues, gender equality, and immigration (Inglehart, 1971, 2017; Norris & Inglehart, 2019). This has resulted in the emergence of a new socio-cultural dimension alongside the traditional socio-economic one, creating ideological cleavages along a Green, Alternative, Liberal (GAL) dimension and a Traditional, Authoritarian, National (TAN) one (Hooghe et al., 2002). Although these dimensions appear separate, there are overlaps, with left-wing individuals favoring liberal values and right-wing individuals favoring traditional or conservative values (Oscarsson et al., 2021).

The expansion of the media entails an increase in opportunities for media choices tied to outlets with distinct political profiles (Bennett & Iyengar, 2008; Skovsgaard et al., 2016; Stroud, 2017). Notably, right-wing alternative news media emphasizes socio-cultural values over socio-economic ones, highlighting issues such as immigration and crime (Holt et al., 2019). Despite these shifts, research tend to rely on socio-economic ideology as a predictor in explaining news exposure and media effects (Dahlgren, 2020; Fletcher et al., 2020). Thus, it is important to take the emerging socio-cultural dimension into consideration, as it may influence people’s perceptions of political and societal issues in different ways compared to the socio-economic dimension (Shehata et al., 2022). To a certain extent, the socio-cultural dimension has been neglected, and that is why this study compares both the socio-cultural and the socio-economic dimensions. It is not far-fetched to assume that the socio-cultural dimension will work as a stronger dispositional as well as susceptibility variable, than the socio-economic one given that: (1) violent crime, and crime in general, is an issue emphasized along the socio-cultural dimension (Inglehart, 1971, 2017), and (2) in the context of Sweden, ideological and political polarization is more stable along the socio-economic dimension compared to the socio-cultural one where there has been a substantial occurrence of ideological polarization (Oscarsson et al., 2021).

Drawing on the DSMM, it is therefore conceivable that ideology works as a dispositional factor, motivating citizens to interpret information about violent crime in line with their existing ideological and societal perceptions (Molden & Higgins, 2012; Stroud, 2010; Valkenburg & Oliver, 2019). Consequently, predisposed ideology is expected to have a dual impact on people’s crime perceptions. Firstly, it serves as an antecedent variable predicting news use. Secondly, it conditions the interpretation of received information, aligning perceptions with individuals’ ideological predispositions (Valkenburg & Peter, 2013).

## Research hypotheses and question

Based on previous research and theories, as outlined above, this study tests whether ideology predicts news exposure and conditions the effects of news use, producing negative or positive perceptions of the development of violent crime in society (Shehata & Strömbäck, 2020; Valkenburg & Oliver, 2019). The following hypotheses are posed.

It is expected that citizens’ predisposed ideology will play a significant role in maintaining and possibly reinforcing citizens’ perceptions of violent crime over time. A comparison between the established socio-economic dimension and the socio-cultural one will make it possible to investigate different ideological dimensions as a driver of citizens’ perceptions.

H1: Citizens’ societal perception of violent crime is influenced by ideology.

If a development of perceptions occurs through a process of selective exposure, the two dimensions should play a key role in what news outlets people consume, and selective news use should influence subsequent perceptions across ideological groups.

H2: The socio-economic dimension of ideology influences citizens’ perception of violent crime through selective news use (mediating effect).

H3: The socio-cultural dimension of ideology influences citizens’ perception of violent crime through selective news use (mediating effect).

To investigate whether the two dimensions play more than a dispositional role in the development of perceptions, this study examines the possible influence of ideology on the relationship between news use and perceptions of violent crime.

H4: Socio-economic ideology moderates the influence of news use by reinforcing ideological differences in perceptions of violent crime (moderation effect).

H5: Socio-cultural ideology moderates the influence of news use by reinforcing ideological differences in perceptions of violent crime (moderation effect).

Since this study examines two ideological dimensions, differences between the two dimensions are examined by asking the question:

RQ1: Are there any differences between the ideological dimensions in how they condition the relationship between news use and perceptions of violent crime?

### The context of Sweden

The Swedish media system is a typical democratic corporatist model (Hallin & Mancini, 2004), dominated by public broadcasting (Ohlsson, 2015). In contrast to other media systems, the opportunity structures in Sweden for ideological media use are relatively scarce. There are few newspapers or television and radio channels with a strong ideological association similar to Fox News, MSNBC, or political talk radio (Bennett & Iyengar, 2008; Skovsgaard et al., 2016). However, there is still ideological content to be found, especially online, through both alternative and mainstream news sites.

The four leading newspapers in Sweden have clear historic political profiles along the left-right dimension. *Aftonbladet* is an independent social democratic tabloid. The tabloid *Expressen* and the broadsheet paper *Dagens Nyheter* both identify as independent liberal newspapers, and *Svenska Dagbladet* is an independent conservative broadsheet newspaper. Most of the ideological content aimed at opinion-building in these newspapers is nowadays isolated to the editorial pages and seldom present in news coverage (Weibull, 2013). Together with public broadcast media, Sveriges Television (SVT1, SVT2) and Sveriges Radio (SR), the commercial television channel TV4 and the included newspapers are the most used news outlets in Sweden according to the annual survey “Media Barometer” (Ohlsson, 2021).

Alternative outlets in Sweden are mostly found online, where ideological content is present in both editorials and in news coverage. Two of the most well-known examples are *Dagens ETC* (left-wing) and *Samhällsnytt*^2^^2^The outlet goes by both *Samhällsnytt and Samnytt* (Widfeldt, 2023). (right-wing), both aiming at opinion-building by publishing more explicit ideological news content (Dahlgren et al., 2019; Weibull et al., 2018). Alternative outlets are important by representing the clearest opportunity for ideological news consumption within the Swedish media system (Skovsgaard et al., 2016).

**Figure 2.**
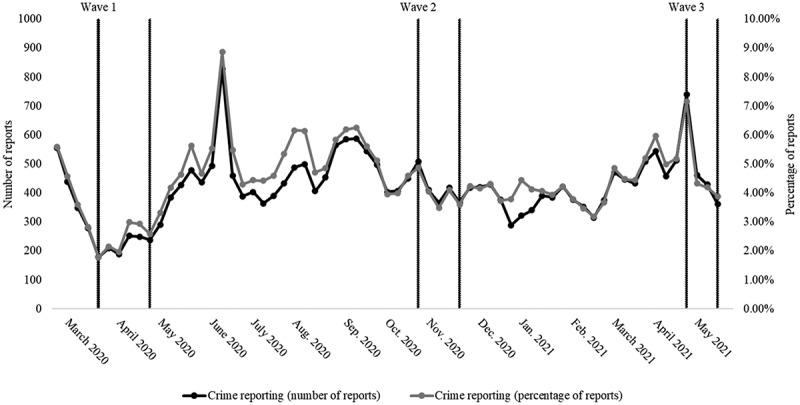
Reporting on violent crime, February 17, 2020–May 17, 2021.

To provide a descriptive picture of the reporting volume of violent crime in Sweden, a search string is constructed to capture content concerning violent crime using the Media Archive.^3^^3^The Media Archive is the largest media repository in the Nordic region, archiving news including print, digital, radio, and television and it spans content from the 1980s onward. By counting all articles and broadcasting news during the study period, Figure 2 illustrates the total number and percentages of violent crime reporting in all the newspapers, both traditional and digital ones, and broadcasting outlets mentioned above.

*Amount and percentage are both shown*. Alternative outlets: *Aktuellt Fokus*, *Aktuellt i Politiken*, *Aktuellt i Politiken web*, *Arbetet web*, *Arbetet*, *Dagens Arena*, *Dagens ETC*, *ETC web*, *Fria Tider*, *Ledarsidorna*, *Nya Tider*, *Nyheter Idag*, and *Samhällsnytt*. Traditional press with a political leaning: *Aftonbladet*, *Aftonbladet web*, *Dagens Nyheter*, *Dagens Nyheter web*, *Expressen*, *Expressen web*, *Svenska Dagbladet*, *Svenska Dagbladet web*, and *Svenska Dagbladet Premium*. Public service and nonpolitical outlets: *SVT Nyheter web* (digital read news), *SR Ekot*, *Rapport*, *Aktuellt*, and *TV4 Nyheterna*. The search engine cannot differentiate between television, radio programs, and specific reports on violent crime. It counts every program mentioning the search terms. Thus, broadcasting media has some skewness compared to other media types, it remains crucial to include them as they are an integral part of the Swedish media landscape. Total amount of items = 623,614. Search terms used: violent crime* OR assault* OR *murder* OR *shooting* OR bombing* OR gang crime* OR gang war* OR “organized* crime*” OR “serious* crime*” OR knife robbery* OR humiliation robbery* OR elderly robbery * OR “fatal* violence*” OR juvenile robbery* OR “armed robbery” OR knife violence* OR firearm violence* OR gang violence* OR “serious* violence*” OR death by shooting* OR violence* OR sexual crime* OR violation of women’s integrity* OR “violence against women” OR manslaughter*.

*Source*: Mediearkivet Retriever

Generally, the reporting on crime during the period of investigation is lowest at Wave 1 of the panel survey used in this study (see method section for more detail). Between Waves 1 and 2 there is generally a higher level of reporting, probably due to the increased number of shootings during spring and autumn in 2020 (TT News Agency, 2021). The highest number of items in comparison to the other two waves is found in the beginning of the last wave. These results were collected in the same week as a very young man was shot in the third-largest city in Sweden, which generated a great deal of publicity (Kjelldén & Gertten, 2021).

In absolute numbers, the traditional mainstream news media reports the majority of crime-related news. In relative terms, however, it is amongst alternative outlets that we find the highest percentage of news items about violent crime, where the right-wing outlets provide most of the news items as shown in Figure A1.

## Data and analytical approach

### Data

In answering the research question and hypotheses outlined above, this study relies on a three-wave panel survey collected in Sweden between March 17, 2020 and May 14, 2021. Data collection was conducted by the Laboratory of Opinion Research (LORE) at the University of Gothenburg, utilizing a probability sample, pre-stratified on age, sex, and education. The study was approved by the Swedish Ethical Review Authority in January 2020 under case number Dnr 2019–04079. In Wave 1 (March 2020), 3,327 respondents were invited to participate, where 2,171 started the survey and 2,058 completed the survey with a 62.7% gross participation rate and 65% net participation rate. In Wave 2 (October 2020), the survey was distributed to 3,134 respondents, of which 1,785 started the survey and 1,700 made a complete response with a 55.1% gross participation rate and 57.6% net participation rate. In Wave 3 (April 2021), 3,010 respondents received the survey, of them 1,608 respondents started the survey, and 1,551 made a complete response with a 52.3% gross participation rate and 55.3% net participation rate.

The goal in a survey like this is of course to ensure that the sample reflects the population, in this case the Swedish population, as closely as possible. Yet, in a panel like this, respondents may be more interested in politics and news than would the average Swede. A comparison between the study sample collected and the Swedish population is shown in Table 1. The panel survey assesses individuals’ perceptions of violent crime, ideological predispositions, news consumption, and other background variables. In total, 1,303 respondents answered all relevant survey items and participated in all three waves.

**Table 1.** Swedish population data from 2020 for age, education level, and sex, compared to the sample data.

	Swedish population		Sample		Sample answering all relevant questions	
	percent	frequency	percent	frequency	percent	frequency
**Age**						
Under 30	13.57	1,408,214	12.53	272	9.42	108
30–39	13.48	1,398,679	14.92	324	12.82	147
40–49	12.54	1,301,462	18.38	399	18.57	213
50–59	12.62	1,310,245	17.23	374	19.18	220
60–69	10.66	1,105,960	19.81	430	21.71	249
70 or Older	12.42	1,288,662	17.13	372	18.31	210
Total	100.00	10,379,295	100.00	2,171	100.00	1,147
**Education**						
Low Education	54.32	680,357	42.29	839	40.84	468
High Education	45.68	572,121	57.71	1,145	59.16	678
Total	100.00	1,252,478	100.00	1,984	100.00	1,146
**Sex**						
Female	49.57	3,818,041	50.25	1,091	48.82	560
Male	50.43	3,884,190	49.75	1,080	51.18	587
Total	100.00	7,702,231	100.00	2,171	100.00	1,147

The table shows Swedish population data from 2020 for age, education level, and sex, compared to the sample data and compared to the sample where respondents answering all relevant questions in all three waves are included. To match the data sets the variable for education has been recoded into two categories: 1. Low Education, which entails “Elementary School-High School” and 2. High Education, which entails “Post High School Education- Postgraduate Education.”

*Sources*: Statistics Sweden and ERC project VARME.

### Measurements

#### Ideological dimensions

To capture both a socio-economic and a socio-cultural dimension, this study uses attitudes toward policy proposals associated with materialistic and post-materialistic values, to construct additive indexes for each ideological dimension (see Shehata et al., 2022). A principal component factor analysis has been employed to identify the possible two dimensions. In accordance with the literature on factor analysis, a Bartlett’s test of sphericity and a Kaiser Meyer Olkin (KMO) test have been conducted to determine the appropriateness of performing a factor analysis (Atan & Kasmin, 2018; Dziuban & Shirkey, 1974).

In line with earlier research, the results exhibit a two-factor solution that corresponds with the ideological dimensions as shown in Tables A3, A4, and A5 in the online supplemental materials (Hooghe et al., 2002). However, item “1. Lower taxes” was removed, as it has equally high loadings on both factor 1 and 2 as shown in Table A5 in the online supplemental materials. A second item, “5. Introduce much harsher prison sentences for criminals,” was deleted for theoretical reasons, namely that it is a question too close to the dependent variable.

The socio-economic dimension consists of three policy proposals, shown in Table A6 in the online supplemental materials, capturing the aspects of traditional socio-economic issues. More specifically, the respondents were asked “What is your opinion with respect to the following proposals?”: (1) “Prohibit profit for companies within the healthcare system, elderly care and education,” (2) “Increase unemployment benefits,” (3) “Reduce income disparities in society.” The proposal is followed by five options for response: (1) “Very good proposal,” (2) “Somewhat good proposal,” (3) “Neither good nor bad proposal,” (4) “Somewhat bad proposal,” (5) “Very bad proposal.” The theoretical range for the index extends from 1 = *left-wing* to 5 = *right-wing*: *M* = 2.31, *SD* = 0.90, Cronbach’s α = 0.66.

The socio-cultural dimension builds on the following three out of eight policy proposals capturing the “new” cultural aspects of socio-cultural issues: (1) “Sweden should reduce the intake number of refugees” (reversed), (2) “Increase the carbon dioxide tax on petrol,” and (3) “Aim for a multicultural society.” The theoretical range of the cultural index also extends between 1 = *liberal* to 5 = *conservative*: *M* = 3.06, *SD* = 1.12, Cronbach’s α = 0.76.

The two scales constructed based on the factor analysis and a Pearson’s *r*, *r* (1297) = .41, *p* < .001, suggest, in line with previous empirical work, that these are two separate dimensions (Hooghe et al., 2002) that to some extent correlates. In the respective index, low values indicate a socio-economic left and socio-cultural liberal leaning, while high values represent a socio-economic right and socio-cultural conservative leaning.

#### News media use

Exposure to frequently used national news outlets is measured by asking the respondents how often they have used different outlets in the past month, with the responses: 1 = “Never,” 2 = “Rarely,” 3 = “1–2 days a week,” 4 = “3–4 days a week,” 5 = “5–6 days a week,” and 6 = “Daily.” The broadcasters included are SVT, SR, and *TV4 Nyheterna*. The newspapers are *Aftonbladet*, *Expressen*, *Dagens Nyheter*, and *Svenska Dagbladet*. For outlets with both online and print formats, respondents’ highest usage frequency is measured as their score. Alternative news includes the following left-wing outlets: *Aktuellt Fokus*, *Arbetet*, *Aktuellt i Politiken*, *Dagens Arena*, and *Dagens ETC;* and right-wing outlets: *Fria Tider*, *Ledarsidorna*, *Nya Tider*, *Nyheter Idag*, *Samhällsnytt*, and *Samtiden*.

#### Ideological news use

This study utilizes an audience profile approach following Fletcher et al. (2020), to explore how individuals distribute their attention across different outlets, creating diverse audience profiles with varying degrees of polarization (Webster & Ksiazek, 2012). Previous scholars have used this approach to measure and study individual-level ideological selective exposure in people’s news diets (Fletcher et al., 2020; Shehata et al., 2022). To examine the role played by news use in relation to perceptions, this study uses the same approach relying on two indexes that identify people’s different ideological news diets. By constructing the two indexes, it is possible to separate between news audience profiles within the socio-economic and the socio-cultural dimensions (Shehata et al., 2022), which is imperative for the aims of this study.

The socio-economic and socio-cultural profile of an outlet’s typical audience is used as a proxy to categorize the outlet’s ideological leaning or slant. The following equation estimates the ideological slant of each outlet by subtracting the ideological mean of the typical user from the sample mean for each ideological dimension (Fletcher et al., 2021):

s=o−p

In this equation, *s* equals the outlet slant (weight), *o* represents the index average of socio-economic and socio-cultural ideology amongst the typical outlet users (using the outlet five or more days a week), and *p* represents the mean ideological leaning of the total sample. This estimates how much the typical audience of each outlet deviates from the population mean along the socio-economic and the socio-cultural dimensions as shown in Appendix A8 in the online supplemental materials. To capture what outlets an individual tends to prefer in both indexes, either left-wing/liberal or right-wing/conservative outlets, each outlet’s ideological slant (s) is multiplied by the frequency of the respondent’s exposure to the outlet (e). This is followed by summarizing the product of s * e across all the outlets (k) (see: Shehata et al., 2022):

$$X_{\mathrm{iw}}=\underset{k}{\sum }s*e$$

This generates a score that captures each individual’s news consumption, where low values capture left-wing consumption profiles and high values right-wing audience profiles along a socio-economic scale. Along the socio-cultural scale low values indicate culturally liberal audience profiles, whereas high values express cultural conservative audience profiles as shown in Appendix A8 in the online supplemental materials.

#### Perceptions of violent crime

In constructing the index for the dependent variable, a battery of three items is used and includes the questions (see Glogger et al., 2023): “In the public debate, there are various statements about crime and criminality. To what extent do you agree with the following statements?” These were followed by the statements: “Violent crime has decreased in Sweden in recent years,” “There is more violent crime in Sweden than in most other countries in the EU,” and “Sweden today has very big problems with violent crime.” The respondents rated their answers on a 7-point scale ranging from 1 = *Not true at all* to 7 = *Completely true*. The questions are designed to capture people’s perceptions about violent crime.

The initial statement was reversed to align with the positive-to-negative dimension observed in the subsequent variables. Reliability coefficients were assessed with Cronbach’s α for each wave: W1 = .78; W2 = .79; W3 = .78. The constructed indexes range from 1 = *positive perception* to 7 = *negative perception* with crime perceptions: W1: *M* = 4.55, *SD* = 1.41; W2: *M* = 4.75, *SD* = 1.42; W3: *M* = 4.77, *SD* = 1.34. Perceptions of violent crime differ among individuals, but its occurrence always poses a societal concern (e.g., O’Connell & Whelan, 1996; Velásquez et al., 2018). The dependent variable in this study aims to capture negative perceptions regarding violent crime, signifying perceptions of its rise, heightened severity in Sweden compared to other countries, and its critical societal impact. Conversely, positive perceptions indicate the absence of observed increases, the perception that Sweden’s situation is not worse than in other countries, and a view that violent crime is not a significant societal problem.

#### Control variables

In the empirical analysis, additional control variables have been used. In Sweden, there is a gender gap in political beliefs, where women are more likely to support left-wing parties, while men are more prone to right-wing ones (Oskarson & Wängnerud, 2013). In turn, the ideological differences between men and women are likely to influence the respondents’ news use and perceptions. The variable for sex is coded 0 = female and 1 = male. Age is also expected to influence the respondents’ news use and have an impact on the dependent variable (Holt et al., 2013). Age is measured and recoded with six categories from under 30” to “70 or above and was rescaled to range from 0 to 1. Education is known for influencing both political behavior (Persson, 2015) and news use (Kwak, 1999), thus education is controlled for. Education is measured on a 9-point scale from 1 = no education to 9 = Postgraduate education, which has also been rescaled to range from 0 to 1. Table A2 in the online supplemental materials summarizes those statistics.

### Analytical approach

**Figure 3.**
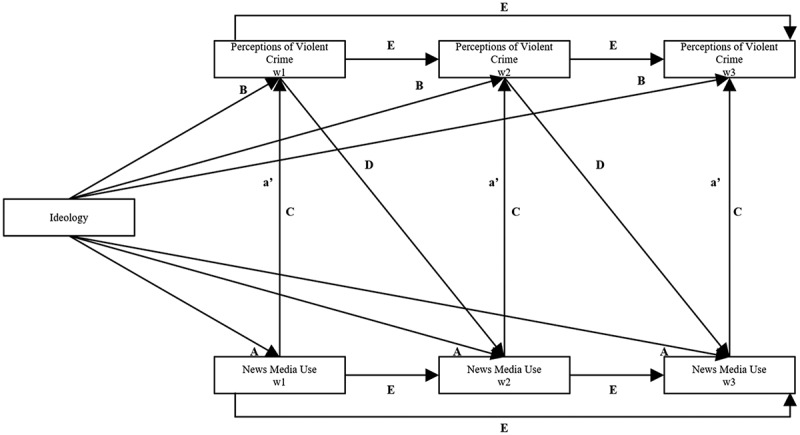
Theoretical model.

To test the DSMM framework in analyzing the influence of ideology on both news use, perceptions of violent crime, and a possible moderating effect of the relationship between news use and perceptions, a lagged panel model, using path analysis, is estimated applying structural equation modeling (SEM). The theoretical model is illustrated in Figure 3.

Path A measures the direct effects of ideology on media use and path B the direct ideological effects on perceptions of violent crime. Path C evaluates the direct effect of media use on perceptions and path D previous perceptions on media use. Path E represents the stability measures over time. Path a′ indicates the mediated total indirect effect of ideology on violent crime perceptions.

Using longitudinal data and the SEM approach enables the observation of direct influences of ideological dimensions and direct/indirect effects of news media use on perceptions of violent crime over the three waves (Acock, 2013). In path A, ideological news exposure is investigated, and in path B direct ideological effects on violent crime perceptions are assessed. Path C measures the direct effect of news use on perceptions, and path D measures previous violent crime perceptions on news use. Path a′ represents the total indirect effects, mediation, of ideology channeled through all possible indirect paths.

In a conventional cross-lagged model, the mediation would start at path A, going from ideology to news use in Wave 1, and path a′ would go from news use to violent crime perceptions in Wave 2. However, in this model, the specific mediation effect is examined within waves, as the news uses variable measures how often the respondent has used the respective outlet during the past month. Drawing on implications of the DSMM, the model should capture the more immediate impact of news use affecting future perceptions. Finally, the effects of crime perceptions are, through path D, influencing news use in the next wave (Valkenburg & Peter, 2013). Thus, earlier influence of news use in previous waves are controlled for throughout the model.

The SEM approach enables and facilitates calculation of the mediating effect of news use on perceptions using direct and indirect effects (Acock, 2013). Leaning on Brambor et al. (2006), possible moderation effects of ideology on the relationship between news use and crime perceptions are assessed separately through ordinary least square regression analysis, focusing on the ideological values of those for whom news use has an effect on crime perceptions.

The two types of modeling allow a comparison of selective exposure and motivated reasoning effects on societal perceptions (Preacher et al., 2007), shedding light on how ideology influences news selection and moderates the influence of news use on crime perceptions over time.

## Results

**Figure 4.**
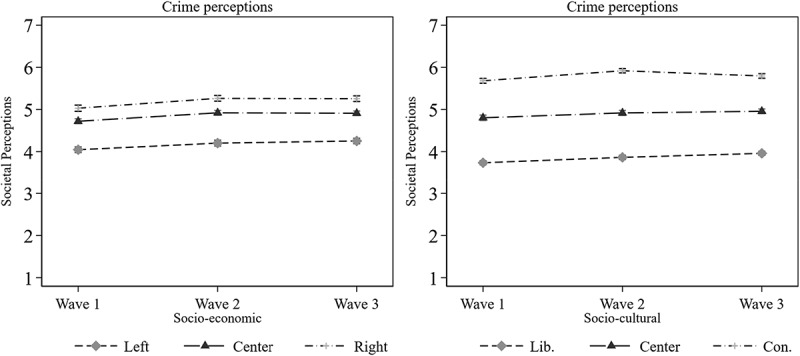
Development of perceptions of violent crime across the socio-economic and the socio-cultural dimensions over time (Means).

In a first step testing the DSMM and the dual role of ideology on media effects, descriptive results of the relation between ideology and crime perception are presented in Figure 4. This is followed by investigating the possible mediated effects through ideological news media use. Then, the conditional effects of ideology on the relation between news use and perceptions are examined. Figure 4 illustrates the mean values of perceptions over the socio-economic and socio-cultural dimensions across three waves.

Ideology, socio-economic and socio-cultural, is divided into three approximately equally sized groups: Left leaning (W1: *n* = 442, W2: *n* = 442, W3: *n* = 607), center leaning (W1: *n* = 512, W2: *n* = 486, W3: *n* = 320), and right leaning (W1: *n* = 345, W2: *n* = 372, W3: *n* = 368). Liberal leaning (W1: *n* = 447, W2: *n* = 534, W3: *n* = 534), center leaning (W1: *n* = 497, W2: *n* = 390, W3: *n* = 380), and conservative leaning (W1: *n* = 356, W2: *n* = 376, W3: *n* = 382). Both the ideological measures are coded, respectively: 1 = left, 2 = center 3 = right, and 1 = culturally liberal, 2 = center, 3 = culturally conservative. Perceptions of violent crime are coded 1 = positive perceptions and 7 = negative perceptions (*n* = 1,241).

In comparing crime perceptions across ideological dimensions, right-leaning and culturally conservative-leaning individuals tend to exhibit more negative perceptions. This is the case over the three waves and the ideological dimensions, yet right-leaning individuals converge with the ideological center in the third wave. This convergence may be facilitated by smaller general differences within the socio-economic dimension compared to the socio-cultural one, coupled with a rapid drop in crime reports at the start of wave 3 as shown previously in Figure 2. However, left-leaning, culturally liberal-leaning, and ideologically center-positioned individuals generally hold fewer negative perceptions—except in wave 3—than their right-leaning and culturally conservative-leaning counterparts. Nevertheless, perception gaps for ideological groups appear more stable from a socio-cultural perspective.

Differences between ideological groups are more pronounced in the socio-cultural dimension than within the socio-economic one. Culturally conservative individuals in the socio-cultural dimension exhibit more negative perceptions of violent crime compared to their liberal counterparts. Contrasted with the socio-economic dimension, the distinctions between socio-economically left-wing and right-wing individuals are smaller than those between culturally liberal and conservative individuals. Individuals in the socio-cultural center tend to be more centered than their socio-economic counterparts, where those in the center tend to lean more toward a right-wing position. The results of Figure 4 support H1 and RQ1, emphasizing perceptual differences tied to ideological leanings (H1) and a stronger ideological impact within the socio-cultural dimension (RQ1).

### Ideological news use

**Figure 5.**
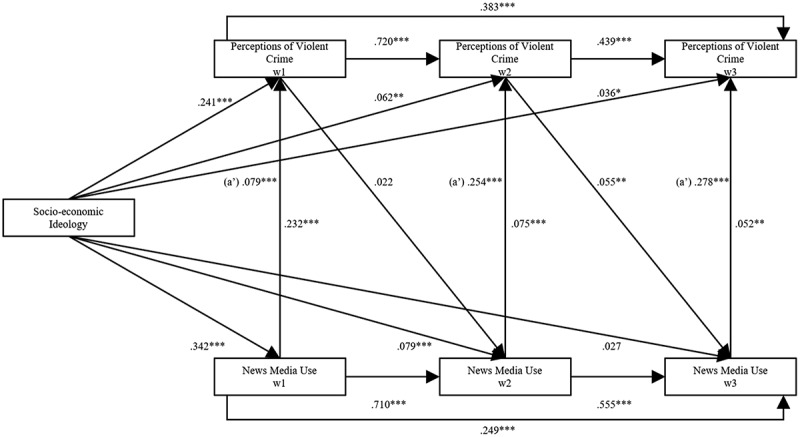
Mediating effects of news media use on perception of violent crime waves 1–3 (Socio-Economic Dimension).

**Figure 6.**
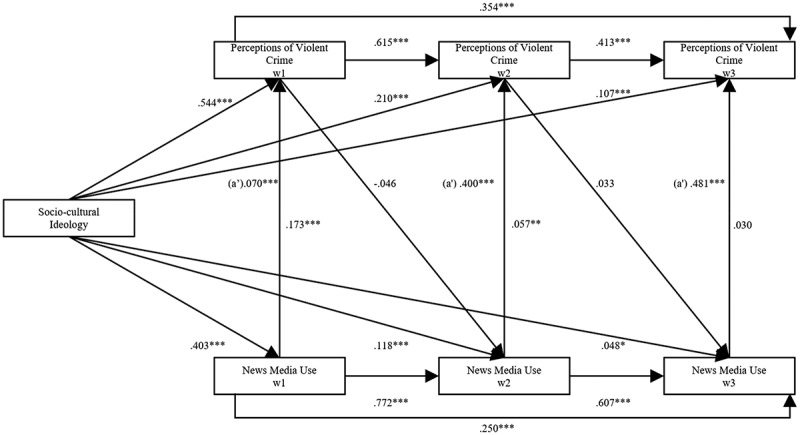
Mediating effects of news media use on perception of violent crime waves 1–3 (Socio-Cultural Dimension).

To understand the dual role played by ideology in perceptions of violent crime, this paper initially investigates whether news use mediates the relation between ideology and perceptions, followed by the possible moderation effects. Before examining the mediating effects of news media use and effects of the ideological dimensions on perceptions, the direct, total indirect, and specific indirect effects need to be presented and analyzed to explore if there may be a mediating relationship between ideology and crime perceptions (Acock, 2013). In Figures 5 and 6, an analysis using structural equation modeling assesses both the direct and total indirect effects.

All estimates in the model are standardized direct effects controlling for sex, age, and education (not shown in the figure). In the model, *a′* represents the mediating total indirect effects of ideology on perceptions of violent crime. Model fit: *X*^2^ (4) = 1.774; root mean square error of approximation (RMSEA) = 0.000; comparative fit index (CFI) = 1.000; Tucker-Lewis index (TLI) = 1.004. *n* = 1,303. Significant levels presented: *p < .05 **p < .01 ***p < .001.

All estimates in the model are standardized direct effects controlling for sex, age, and education (not shown in the figure). In the model, *a′* represents the mediating total indirect effects of ideology on perceptions of violent crime. Model fit: *X*^2^ (4) = 1.774; RMSEA = 0.014; CFI = 1.000; TLI = 0.999. *n* = 1,303. Significant levels presented: *p < .05 **p < .01 ***p < .001.

Firstly, the direct effects between ideology and crime perceptions are analyzed. The results from Figure 5 indicate statistically significant direct positive effects between socio-economic ideology and perceptions of violent crime in Wave 1: β = .241, *p* < .001; Wave 2: β = .062, *p* < .001; and Wave 3: (β = .036A, *p* = .044. Figure 6 exhibit statistically significant direct positive effects between socio-cultural ideology and perceptions of violent crime in Wave 1: β = .544, *p* < .001; Wave 2: β = .210, *p* < .001; and in Wave 3: β = .107, *p* < .001. These initial results strengthen the findings in Figure 4. Right-wing and conservative people tend to have more negative perceptions than left-wing and liberal people.

Secondly, the direct effects of ideology on news use and the lagged effects of crime perceptions on subsequent news use are analyzed. Figure 5 displays significant effects between socio-economic ideology and news media use in Wave 1: β = .342, *p* < .001 and in Wave 2: β = .079, *p* < .001; however, not for Wave 3. Turning to the direct effect of socio-cultural ideology on news use in Figure 6, the effects are found to be statistically significant in Wave 1: β = .403, *p* < .001; Wave 2: β = .118, *p* < .001; and in Wave 3: β = .048, *p* = .011). Examining the lagged direct effects of crime perception on subsequent news use, path D, controlling for habitual news use (Shehata et al., 2022; Stroud, 2017), there is a lack of statistically significant effects of prior crime perceptions on news use except for the socio-economic dimension in Wave 3: β = .055, *p* = .055. When considered together, the results of the direct effects indicate that ideological news use is present, which lends partial support to H2 and H3.

Thirdly, the relation concerning the direct effects between news use and perceptions are examined. Analyzing the socio-economic dimension, results demonstrate significant direct positive effects between news use and crime perceptions in Wave 1: β = .232, *p* < .001; Wave 2: β = .075, *p* < .001; and in Wave 3: β = .052, *p* = 003; whereas in the socio-cultural dimension only Wave 1: β = .173, *p* < .001 and Wave 2: β = .057, *p* = .002 are significant. These results indicate consistent media effects in a lagged model over three waves, suggesting that both right-wing news media and culturally conservative consumption led people to adopt more negative crime perceptions.

### Mediating ideology

As the results of the direct effects between the necessary variables are calculated and presented, we can now continue to first calculate the total indirect effects^4^^4^The total indirect effects are calculated using all possible indirect paths that ideology may be channeled through. Multiplying each direct effect a long a path gives a product of an indirect effect. These indirect effects are then added together throughout the waves resulting in the total indirect effect. For example, in wave 2.Path 1. Socio-economic Ideology × Belief w1 × Belief w2 = indirect effectPath 2. Socio-economic Ideology × Media w1 × Belief w1 × Belief w2 = indirect effectPath 3. Socio-economic Ideology × Media w1 × Media w2 × Belief w2 = indirect effectPath 4. Socio-economic Ideology × Media w2 × Belief w2 = indirect effectPath 5. Socio-economic Ideology × Belief w1 × Media w2 × Belief w2 = indirect effectTables of all calculated indirect and total indirect effects for all three waves and two ideological dimensions, are available upon request from the author. of ideology on crime perception. Secondly, the specific individual indirect effect of ideology through news use to perceptions of violent crime is calculated. Calculating the specific indirect effects is done using the Stata command *nlcom*, which is a post-estimation command calculating the functions of model parameters providing standard errors and confidence intervals of the estimates. Thus, the command provides us with both the specific indirect effect and the statistical significance of that effect (Acock, 2013).

The total indirect effect in Wave 1 is also the specific indirect effect as we in Wave 1 only use the three variables. The result of socio-economic ideology on perceptions of violent crime through news media use is statistically significant, β = .079, *p* < .001, which implies that the effect of ideology on perceptions is partially mediated through news media use in Wave 1. In Wave 2 the total indirect effect, β = .254, *p* < .001, is statistically significant and the specific path, through news media use, is also statistically significant: β = .006, *p* = .007). The specific indirect effect demonstrates a partial mediation of news media use on the relation between ideology and crime perceptions. The result suggests that predisposed ideological perceptions of violent crime is to some extent mediated through news use. In Wave 3, the total indirect effect of ideology is statistically significant: β = .278, *p* = .008. However, the specific indirect effect of socio-economic ideology through news use is not. This result indicates that other specific paths through which socio-economic ideology is mediated appear to be more important than news media use in Wave 3.

Observing the socio-cultural dimension, the total indirect effect of ideology on crime perceptions is statistically significant, and only follows the path through news use, β = .070, *p* < .001, implying a partial mediation of news use on perceptions. In Wave 2, the total indirect effect of socio-cultural ideology on perceptions of violent crime is statistically significant: β = .400, *p* < .001. Estimating the specific indirect effect of ideology through news use on crime perceptions, a partial mediation effect is found, accounted for by news use: β = .007, *p* = .008. A statistically significant total indirect effect of ideology on crime perception is found in Wave 3: β = .481, *p* < .001. Yet, as the hypothesized specific indirect effect running through news use lacks statistical significance, the substantial part of the mediation effect is likely accounted for by other paths in the model.

The specific mediating results, where the effect of ideology on crime perceptions is mediated through news use, are substantially smaller than the total indirect effects. This is because the specific indirect effects are, in every wave, under control for the lagged dependent variable as well as for other control variables and factors which reduces the values of all direct effects throughout the two models. However, because some variables (e.g. at wave 1) are measured simultaneously, caution is warranted, as the mediation effects cannot per se be assumed to reflect a unidirectional influence. For instance, a reverse direction whereby beliefs or media use in wave 1 shape ideology is clearly a possibility that should be noted. Yet, such alternative directions seem unlikely in this case given the theoretical framework and supporting lagged effects included in the model (Hayes et al., 2024).

Nevertheless, results indicate that both within the socio-economic and socio-cultural dimensions, significant indirect effects in the model influence the focal relationship, where news use to some extent does play a significant role in shaping perceptions of violent crime in waves 1 and 2. These results lend some support for both H2 and H3 and uncovers partial mediating effects of ideological news use, leading to specific perceptions.

### Ideological moderation

This study hypothesizes that ideology moderates the effect of news use on perceptions, meaning that the relationship between news consumption and perceptions of crime is conditional on ideological positioning. The interaction between news use and socio-economic ideology in wave 1, β = −0.043, *p* = .079, and wave 2, β = 0.005, *p* = .823, as well as socio-cultural ideology in wave 1, β = −0.014, *p* = .338, and wave 2, β = 0.006, *p* = .679, on crime perceptions indicate non-significant moderation effects of the two ideology dimensions. Following Brambor et al. (2006), the marginal effects across different values of the moderating variable, ideology, was calculated.

The overarching evidence of conditional effects of ideology on the relationship between news use and crime perception, are absent or at best modest. For example, in wave 1, the analysis suggests that amongst respondents with left-wing, somewhat left-wing, or centrist ideologies, higher consumption of right-wing news is associated with more negative perceptions of crime. Even though the results indicate tendencies toward certain effects within some ideological groups as shown in Table A9 in the online supplemental materials, the effects are weak, and the differences are not statistically significant. Therefore, the results lend little support to both H4 and H5.

## Conclusion

This study set out to test whether political ideology, as a dispositional factor, both predicts news exposure and conditions the influence of news use on perceptions of violent crime in Sweden. Overall, the findings show partial support for the assumptions of the Differential Susceptibility to Media Effects Model (Valkenburg & Oliver, 2019; Valkenburg & Peter, 2013; Valkenburg et al., 2016), illustrating that media effects on perceptions are entangled with individual-level predispositions. However, the evidence for the hypothesized dual role of ideology, particularly its moderating function, proved weaker than anticipated.

First, the results demonstrate that both socio-economic and socio-cultural ideology directly predict perceptions of violent crime. Across all three waves right-wing and culturally conservative individuals reported more negative views of the crime development compared to those on the economically left and cultural liberal side of the dimensions (Gerber & Jackson, 2016; Jost et al., 2008). Moreover, ideology also shapes news consumption, where participants were more likely to seek out outlets in line with their ideological predispositions (Knobloch-Westerwick, 2014; Stroud, 2010, 2017), confirming the selective exposure mechanism suggested by previous research.

Second, a partial mediating effect of ideological news use on crime perceptions was identified. In the first and second waves, the influence of socio-economic and socio-cultural ideology on crime perceptions was in part channeled through news consumption, indicating that people’s news media choices, guided by ideology, slightly strengthened their existing crime perceptions (Valkenburg & Peter, 2013; Valkenburg et al., 2016).

Third, and more unexpectedly, limited support was found for the idea that ideology moderates the impact of news consumption on crime perceptions (Valkenburg & Oliver, 2019; Valkenburg & Peter, 2013). Interaction effects between ideological predisposition and ideological news use were non-significant. This suggests that while ideology does influence people’s news consumption, thereby indirectly contributing to crime perceptions, it does not consistently impact media effects once news exposure has occurred (Nickerson, 1998; Taber & Lodge, 2006). Instead, the data point to a subtle and weak pattern in which certain groups, particularly those who do not see crime as a major concern, are more susceptible to negative news portrayals, whereas those already holding strong concerns about crime, often people on the right or with more conservative cultural leanings (Gerber & Jackson, 2016), are less influenced (Malka et al., 2014; Sibley et al., 2012).

These findings offer insights into the complexities of media effects in a high-choice environment. They confirm that ideology predicts news consumption and shape how individuals perceive societal issues. At the same time, the anticipated conditioning role of ideology received only very limited empirical support, implying that motivated reasoning may be more nuanced than directly strengthening news effects by individuals predisposed ideological views (Molden & Higgins, 2012). Nevertheless, results imply that ideological news consumption shape citizens’ perceptions of societal issues like violent crime, which possibly may lead to biased perceptions of policy (Druckman & McGrath, 2019). This could undermine citizens’ ability to hold elected leaders accountable for their past and future policies (Mutz, 1998). It also appears that more individual modeling, taking non-media variables into consideration, gives a broader understanding of how media effects emerge in a more individualized media environment (Valkenburg & Peter, 2013; Valkenburg et al., 2016).

While this study emphasizes ideology as a dispositional variable, it is essential to acknowledge the potential relevance of other variables. Variables such as local and national crime rates, personally mediated victimization experiences, and income level (Roche et al., 2016) may impact perceptions of violent crime. Future research should explore these variables to better understand the significance of dispositional variables in shaping crime perceptions (Valkenburg & Peter, 2013; Valkenburg et al., 2016).

Regarding news media use, the results of the model demonstrate statistically significant effects of news use on perceptions of violent crime. In a socio-economic setting, this effect is stable throughout the three waves, and for the socio-cultural context the same result is found in waves 1 and 2. It is worth mentioning that the current models used in the analysis subjects the results to a robust test by controlling for a lagged variable in each wave. Despite these hard tests of the models, the results are still statistically significant. The results presented imply that neither in modeling on the socio-economic nor on the socio-cultural ideological dimension, are there any significant impact of prior perceptions of violent crime on subsequent news use (Path D). However, it is not surprising that negative perceptions of violent crime do not drive individual news selectivity. News use is largely an endower of routine where short-term changes in perceptions of a single societal issues, such as crime, hardly would outweigh habitual ideological news selectivity (Path A) (Shehata et al., 2022; Stroud, 2017).

The findings of this study only provide partial support for the assumptions of the DSMM, indicating that ideology functions as a predictor of news exposure and crime perceptions over time but only weakly implies that ideology conditions the relation between news use and crime perception (Valkenburg & Peter, 2013). However, this does not disqualify the analysis of dispositional variables. Future research should, when studying media effects in a high-choice media environment, expand on additional dispositional variables as more than one component of a larger dynamic process, by which identities, perceptions, beliefs, and behaviors influence both news media use and media effects (Valkenburg & Peter, 2013; Valkenburg et al., 2016).

There are always limitations when using observational data. First, it should be noted that the three-wave panel study was conducted during the COVID-19 pandemic. This could potentially mean that people’s crime perceptions were affected by the pandemic. For example, there may have been less reporting on crime during this period in favor of stories about COVID-19, making crime less salient than it otherwise would have been, producing different results than those obtained in a study carried out during normal circumstances. Second, while using panel data makes it easier to identify causal-like effects, caution is still warranted in making any strong causal claims about the relationship between our variables, as controlling for all relevant third variables in multiple contexts is impossible. A third limitation is that this paper does not investigate the actual content of the news media that the respondents consume. This fact makes it difficult to draw any strong conclusions about the type of information people are exposed to and how they actually interpret it. However, given the general negative reporting on crime and the descriptive statistics for violent crime reporting, it provides some indications about what the content may be in the outlets people consume. In addition, existing studies examine the relationship between social media and fear of crime (e.g. Intravia et al., 2017), while this study specifically focuses on mainstream and alternative news media. This choice introduces additional limitations: (1) The study exclusively explores the impact of news media consumption, excluding other types of media use; and, (2) Use of social media is not investigated, which leaves out possible technological effects on behavior such as algorithmic selective exposure (e.g. Ohme, 2021). However, news media use through traditional outlets and platforms (online and offline) on television, radio, and in newspapers still remains the predominant means for news consumption amongst Swedish citizens (Internetstiftelsen, 2022).

Concerning ideology shaping party systems, policy issues, voter behavior, news media use, and public opinion (Holmberg & Oscarsson, 2015; Oscarsson, 2020), socio-cultural ideology emerges in this study as a significant predictor of selective exposure and perceptions of violent crime. While socio-economic ideology still holds prominence within the research field, findings in line with earlier research indicate that the socio-cultural counterpart seems more adept at predicting certain societal issues (Glogger & Shehata, 2022). When studying socio-economic and social-cultural ideology, future research should explore other topics and time periods to see whether these dimensions remain distinct or begin to overlap. Testing the socio-cultural dimension in different political and societal contexts may help clarify the underlying theoretical assumptions about the influence of socio-cultural ideology apply to various media and political systems.

Despite the mentioned limitations, this study unveils important implications, contributing to research on media effects and motivated reasoning, stressing the importance of further research on individual non-media variables when examining media effects in the current media environment.

## Supplementary Material

Supplemental Material
