# Supplementary material for: News Media Use and Crime Perceptions: The Dual Role of Ideology
Source: Mass Commun Soc. 2025 Mar 7;28(3):511–29. doi: 10.1080/15205436.2025.2471866 (PMC12011023; doi:10.1080/15205436.2025.2471866)
Supplement: Supplemental Material [file HMCS_A_2471866_SM9131.docx]

# Appendix

Figure A1. Ideological reporting on violent crime, February 17, 2020 - May 17, 2021 (Amount of Reporting and Percent).

**
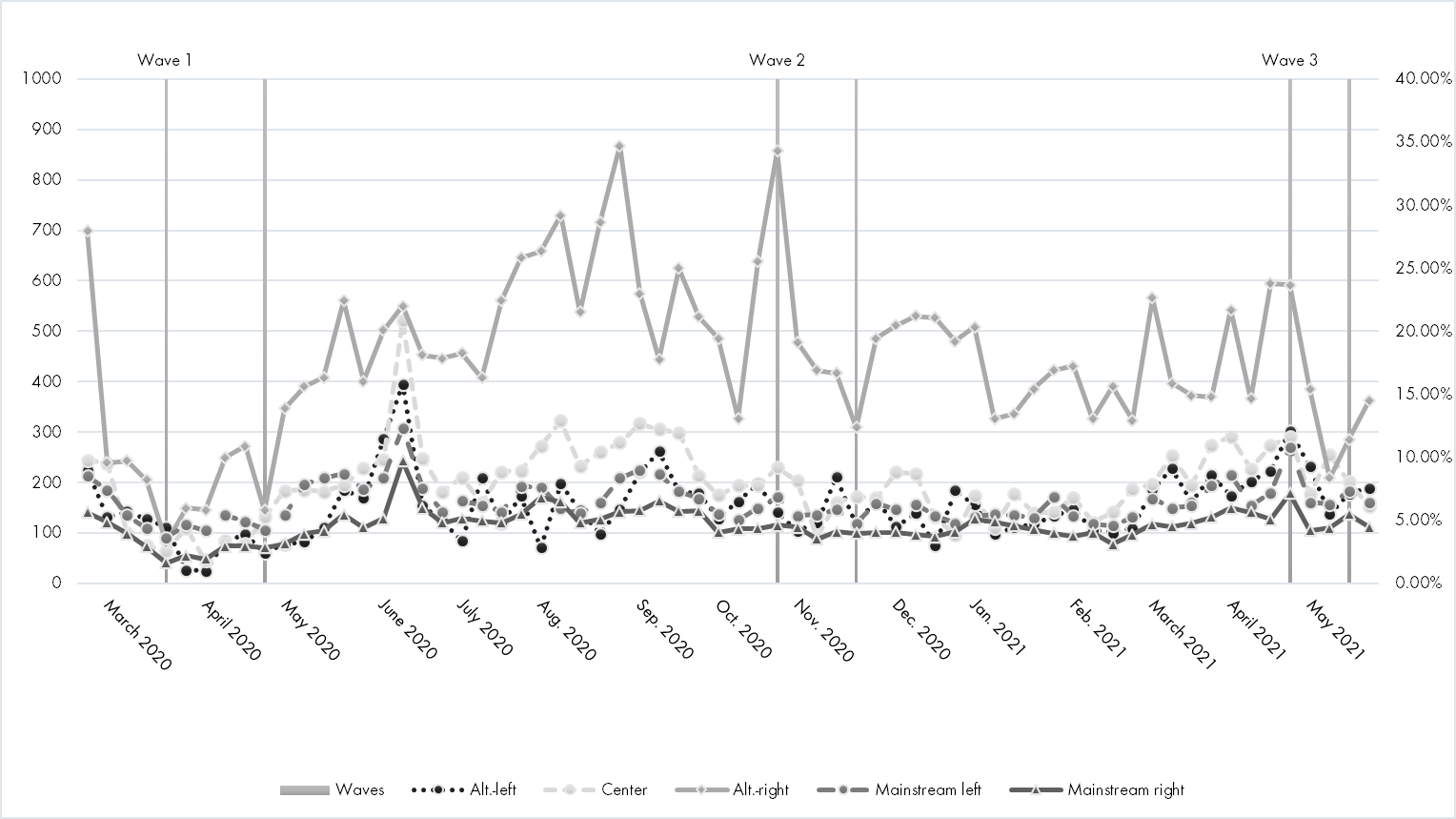
**

*Note:* The categories are divided as follows: Left-wing alternative outlets: *Aktuellt Fokus*, *Aktuellt i Politiken*, *Aktuellt i Politiken web*, *Arbetet web*, *Arbetet*, *Dagens Arena*, *Dagens ETC*, and *ETC web*. Left-wing mainstream outlets: *Aftonbladet and Aftonbladet web*. Center outlets: *SVT Nyheter web* (digital read news), *SR Ekot*, *Rapport*, *Aktuellt*, and *TV4 Nyheterna*. Right-wing mainstream outlets: *Dagens Nyheter*, *Dagens Nyheter web*, *Expressen*, *Expressen web*, *Svenska Dagbladet*, *Svenska Dagbladet web*, and *Svenska Dagbladet Premium*. Right-wing alternative outlets: *Fria Tider*, *Ledarsidorna*, *Nya Tider*, *Nyheter Idag*, and *Samhällsnytt*. The categorization stems from the outlets’ own political identification and public service is deemed to be in the center as it is their obligation to be as objective as possible (Riksrevitionen, 2020). Total amount of items = 623 614. Search terms used: *våldsbrott* OR misshand* OR våldt* OR mörd* OR *mord* OR *skjutning* OR sprängning* OR gängkrim* OR gängkrig* OR “organiserad* brottsl*” OR “grov* brottsl*” OR knivrån* OR förnedringsrån* OR åldringsrån* OR “dödl* våld*” OR ungdomsrån* OR “väpnat rån” OR knivvåld* OR skjutvapenvåld* OR gängvåld* OR “grov* våld*” OR dödsskjutning* OR våldt* OR sexualbrott* OR kvinnofridskränk* OR “våld mot kvinnor” OR dråp*.*

*Source*: Mediearkivet Retriever

Table A2. Descriptive statistics by wave (mean, standard deviation and number of observations).

| **Variable** | **N** | **Mean** | **Std. dev.** | **Min** | **Max** |
| --- | --- | --- | --- | --- | --- |
|  |  |  |  |  |  |
| **Perceptions of violent crime** |  |  |  |  |  |
| Wave 1 | 1,303 | 4.575 | 1.423 | 1 | 7 |
| Wave 2 | 1,303 | 4.777 | 1.430 | 1 | 7 |
| Wave 3 | 1,303 | 4.793 | 1.343 | 1 | 7 |
|  |  |  |  |  |  |
| **Socio-economic media use** |  |  |  |  |  |
| Wave 1 | 1,248 | 1.127 | 1.083 | -1.747 | 5.120 |
| Wave 2 | 1,271 | 1.075 | 1.042 | -2.206 | 5.876 |
| Wave 3 | 1,260 | 1.025 | 1.026 | -1.943 | 5.284 |
|  |  |  |  |  |  |
| **Socio-cultural media use** |  |  |  |  |  |
| Wave 1 | 1,248 | 2.711 | 1.692 | -1.505 | 8.465 |
| Wave 2 | 1,271 | 2.544 | 1.597 | -1.491 | 8.339 |
| Wave 3 | 1,260 | 2.501 | 1.571 | -1.310 | 8.920 |
|  |  |  |  |  |  |
| **Socio-economic ideology** | 1,299 | 2.311 | 0.899 | 1 | 5 |
| **Socio-cultural ideology** | 1,300 | 3.064 | 1.115 | 1 | 5 |
| **Sex** | 1,303 | 0.517 | 0.499 | 0 | 1 |
| **Age** | 1,303 | 0.586 | 0.313 | 0 | 1 |
| **Education** | 1,251 | 0.561 | 0.254 | 0 | 1 |
|  |  |  |  |  |  |
| **News outlets** |  |  |  |  |  |
| **Aftonbladet** |  |  |  |  |  |
| Wave 1 | 1,288 | 3.332 | 1.998 | 1 | 6 |
| Wave 2 | 1,297 | 3.272 | 2.001 | 1 | 6 |
| Wave 3 | 1,291 | 3.222 | 1.994 | 1 | 6 |
|  |  |  |  |  |  |
| **Expressen** |  |  |  |  |  |
| Wave 1 | 1,285 | 2.757 | 1.850 | 1 | 6 |
| Wave 2 | 1,295 | 2.646 | 1.825 | 1 | 6 |
| Wave 3 | 1,294 | 2.588 | 1.799 | 1 | 6 |
|  |  |  |  |  |  |
| **Dagens Nyheter** |  |  |  |  |  |
| Wave 1 | 1,277 | 2.468 | 1.814 | 1 | 6 |
| Wave 2 | 1,290 | 2.328 | 1.747 | 1 | 6 |
| Wave 3 | 1,285 | 2.285 | 1.753 | 1 | 6 |
|  |  |  |  |  |  |
| **Svenska Dagbladet** |  |  |  |  |  |
| Wave 1 | 1,273 | 2.044 | 1.502 | 1 | 6 |
| Wave 2 | 1,292 | 2.001 | 1.493 | 1 | 6 |
| Wave 3 | 1,283 | 1.917 | 1.442 | 1 | 6 |
|  |  |  |  |  |  |
| **Swedish public broadcasting (SVT)** |  |  |  |  |  |
| Wave 1 | 1,301 | 4.284 | 1.763 | 1 | 6 |
| Wave 2 | 1,299 | 4.018 | 1.815 | 1 | 6 |
| Wave 3 | 1,299 | 4.025 | 1.826 | 1 | 6 |
|  |  |  |  |  |  |
| **Swedish public radio (SR)** |  |  |  |  |  |
| Wave 1 | 1,287 | 3.841 | 1.915 | 1 | 6 |
| Wave 2 | 1,298 | 3.705 | 1.942 | 1 | 6 |
| Wave 3 | 1,296 | 3.633 | 1.926 | 1 | 6 |
|  |  |  |  |  |  |
| **Alternative left-wing news use** |  |  |  |  |  |
| Wave 1 | 1,297 | 1.581 | 1.048 | 1 | 6 |
| Wave 2 | 1,294 | 1.491 | 0.957 | 1 | 6 |
| Wave 3 | 1,290 | 1.481 | 0.933 | 1 | 6 |
|  |  |  |  |  |  |
| **Alternative right-wing news use** |  |  |  |  |  |
| Wave 1 | 1,294 | 1.770 | 1.391 | 1 | 6 |
| Wave 2 | 1,295 | 1.563 | 1.182 | 1 | 6 |
| Wave 3 | 1,289 | 1.532 | 1.156 | 1 | 6 |
|  |  |  |  |  |  |

*Note:* The table shows descriptive statistics for all variables used in the analysis.

Table A3. KMO and Bartlett’s tests.

| KMO - sampling adequacy |  | .820 |
| --- | --- | --- |
| Bartlett’s test of sphericity | Chi-square | 3201.604 |
|  | df | 28 |
|  | Sig. | .000 |

*Note:* The table presents the results from KMO and Bartlett’s tests. The variables used are: 1. lower taxes, 2. distribution of profits should not be allowed within state-funded healthcare and schools or other public services, 3. raise unemployment benefits, 4. accept fewer refugees into Sweden, 5. introduce much harsher prison sentences for criminals, 6. raise the carbon dioxide tax on petrol, 7. reduce income disparities in society, and 8. aim for a multicultural society. N = 1 303.

Table A4. Initial Eigenvalues.

| Component | Eigenvalue | Difference | Proportion | Cumulative |
| --- | --- | --- | --- | --- |
| 1 | 3.52091 | 2.26961 | 0.4401 | 0.4401 |
| 2 | 1.25130 | 0.48956 | 0.1564 | 0.5965 |
| 3 | 0.76174 | 0.12471 | 0.0952 | 0.6917 |
| 4 | 0.63703 | 0.04431 | 0.0796 | 0.7714 |
| 5 | 0.59272 | 0.09171 | 0.0741 | 0.8455 |
| 6 | 0.50101 | 0.04872 | 0.0626 | 0.9081 |
| 7 | 0.45229 | 0.16929 | 0.0565 | 0.9646 |
| 8 | 0.28300 | . | 0.0354 | 1.0000 |

*Note:* Likelihood-ratio (LR) test: independent vs. saturated: chi2(28) = 3204.11, Prob > chi2 = 0.0000

Table A5. Rotated factor loadings.

| Variable | Factor1 | Factor2 |
| --- | --- | --- |
| 1. Lower taxes | 0.5054 | 0.5642 |
| 2. Distribution of profits should not be allowed within state-funded healthcare and schools or other public services | 0.0318 | 0.7977 |
| 3. Raise unemployment benefits | 0.1186 | 0.6942 |
| 4. Accept fewer refugees into Sweden | 0.8590 | 0.1475 |
| 5. Introduce much harsher prison sentences for criminals | 0.7474 | 0.0492 |
| 6. Raise the carbon dioxide tax on petrol | 0.6668 | 0.1999 |
| 7. Reduce income disparities in society | 0.3158 | 0.7354 |
| 8. Aim for a multicultural society | 0.7516 | 0.2331 |

*Note:* LR test: independent vs. saturated: chi2(28) = 3204.11, Prob > chi2 = 0.0000

Table A6. Policy proposals.

| Translation | Response |
| --- | --- |
| Lower taxes | (1) Very good proposal  (2) Fairly good proposal  (3) Neither good nor bad proposal  (4) Fairly bad proposal  (5) Very bad proposal |
| Prohibit distribution of profits in tax-financed healthcare, schools or other public services | (1) Very good proposal  (2) Fairly good proposal  (3) Neither good nor bad proposal  (4) Fairly bad proposal  (5) Very bad proposal |
| Raise unemployment benefits (unemployment insurance fund) | (1) Very good proposal  (2) Fairly good proposal  (3) Neither good nor bad proposal  (4) Fairly bad proposal  (5) Very bad proposal |
| Accept fewer refugees into Sweden | (1) Very good proposal  (2) Fairly good proposal  (3) Neither good nor bad proposal  (4) Fairly bad proposal  (5) Very bad proposal |
| Introduce much harsher prison sentences for criminals | (1) Very good proposal  (2) Fairly good proposal  (3) Neither good nor bad proposal  (4) Fairly bad proposal  (5) Very bad proposal |
| Raise the carbon dioxide tax on petrol | (1) Very good proposal  (2) Fairly good proposal  (3) Neither good nor bad proposal  (4) Fairly bad proposal  (5) Very bad proposal |
| Reduce income disparities in society | (1) Very good proposal  (2) Fairly good proposal  (3) Neither good nor bad proposal  (4) Fairly bad proposal  (5) Very bad proposal |
| Aim for a multicultural society | (1) Very good proposal  (2) Fairly good proposal  (3) Neither good nor bad proposal  (4) Fairly bad proposal  (5) Very bad proposal |

*Note:* The table displays the full wording and answering options to the eight proposals used to build a socio-economic and a socio-cultural index.

Table A7. Swedish population data from 2020 for age, education level, and sex, compared to the sample data

|  | Swedish population | |  | Sample | |  | Sample answering all relevant questions | |
| --- | --- | --- | --- | --- | --- | --- | --- | --- |
|  | percent | frequency |  | percent | frequency |  | percent | frequency |
| **Age** |  |  |  |  |  |  |  |  |
| Under 30 | 13.57 | 1,408,214 |  | 12.53 | 272 |  | 9.42 | 108 |
| 30-39 | 13.48 | 1,398,679 |  | 14.92 | 324 |  | 12.82 | 147 |
| 40-49 | 12.54 | 1,301,462 |  | 18.38 | 399 |  | 18.57 | 213 |
| 50-59 | 12.62 | 1,310,245 |  | 17.23 | 374 |  | 19.18 | 220 |
| 60-69 | 10.66 | 1,105,960 |  | 19.81 | 430 |  | 21.71 | 249 |
| 70 or Older | 12.42 | 1,288,662 |  | 17.13 | 372 |  | 18.31 | 210 |
| Total | 100.00 | 10,379,295 |  | 100.00 | 2,171 |  | 100.00 | 1,147 |
|  |  |  |  |  |  |  |  |  |
| **Education** |  |  |  |  |  |  |  |  |
| Low Education | 54.32 | 680,357 |  | 42.29 | 839 |  | 40.84 | 468 |
| High Education | 45.68 | 572,121 |  | 57.71 | 1,145 |  | 59.16 | 678 |
| Total | 100.00 | 1,252,478 |  | 100.00 | 1,984 |  | 100.00 | 1,146 |
|  |  |  |  |  |  |  |  |  |
| **Sex** |  |  |  |  |  |  |  |  |
| Female | 49.57 | 3,818,041 |  | 50.25 | 1,091 |  | 48.82 | 560 |
| Male | 50.43 | 3,884,190 |  | 49.75 | 1,080 |  | 51.18 | 587 |
| Total | 100.00 | 7,702,231 |  | 100.00 | 2,171 |  | 100.00 | 1,147 |
|  |  |  |  |  |  |  |  |  |

Note: The table shows Swedish population data from 2020 for age, education level, and sex, compared to the sample data and compared to the sample where respondents answering all relevant questions in all three waves are included. To match the data sets the variable for education have been recoded into two categories: 1. Low Education, which entails “Elementary School-High School” and 2. High Education, which entails “Post High School Education- Postgraduate Education”.

*Sources*: Statistics Sweden and ERC project VARME.

Table A8. Ideological leaning of Swedish Media Outlets and Daily Reach of Mainstream and Public Broadcast 2020

| **Outlet** | **Left-Right leaning** | **GAL/TAN leaning** | **Daily Reach (percent)** |
| --- | --- | --- | --- |
| Broadcast |  |  |  |
| SVT | -0.0185375 | 0.036278 | 53 |
| SR | -0.0361421 | -0.0747352 | 41 |
| TV4 | 0.1136143 | 0.2998197 | 39 |
| Tabloid |  |  | 27 |
| Aftonbladet | -0.0750029 | 0.1262856 |  |
| Expressen | 0.1870954 | 0.3464715 |  |
| Broadsheet |  |  | 35 |
| Dagens Nyheter | -0.1447022 | -0.4020245 |  |
| Svenska Dagbladet | 0.5572376 | 0.2620645 |  |
| Alternative news media |  |  | N/A |
| Alternative Left-wing | -0.4370654 | -0.1956336 |  |
| Alternative Right-wing | 0.3463914 | 0.6897151 |  |
|  |  |  |  |

Note: The ideological leaning, or slant, is calculated by subtracting the average of the socioeconomic/sociocultural index among regular outlet users (five or more days a week) from the average of the overall sample along the socioeconomic/sociocultural index. This provides estimates of each outlet’s regular audience deviation from the population along the socioeconomic/sociocultural index. Low values indicate left/GAL leaning and high values indicate right/TAN leaning. The results of Daily Reach in the table, refers to the proportion of people consuming news in each medium in traditional or digital form. The sample in the survey is drawn from the Swedish population register (SPAR) and a total of 6 018 randomly selected people answered the survey. The Media Barometer reflects traditional media use throughout the year and on all seven days of the week. The collection period for the Media Barometer 2020 ran from February to June and August to December. Daily Reach of Alternative news use is not measured. The category *Tabloid* entails Aftonbladet and Expressen, *Broadsheet* entails Dagens Nyheter and Svenska Dagbladet The category *Alternative Left-wing* entails ETC, Aktuellt i Politiken, Feministiskt Perspektiv; Dagens Arena, Arbetet, Fria tidningen, and Aktuellt Fokus. *Alternative Right-wing* entails Samhällsnytt, Nya Tider, Nyheteridag, Fridatider, Samtiden, and Ledarsidorna. N=6 018.

Data sources: ERC project VARME and The Media Barometer 2020, p. 93 (Nordicom, University of Gothenburg).

Figure A9. Moderating effects of ideology (marginal effects)


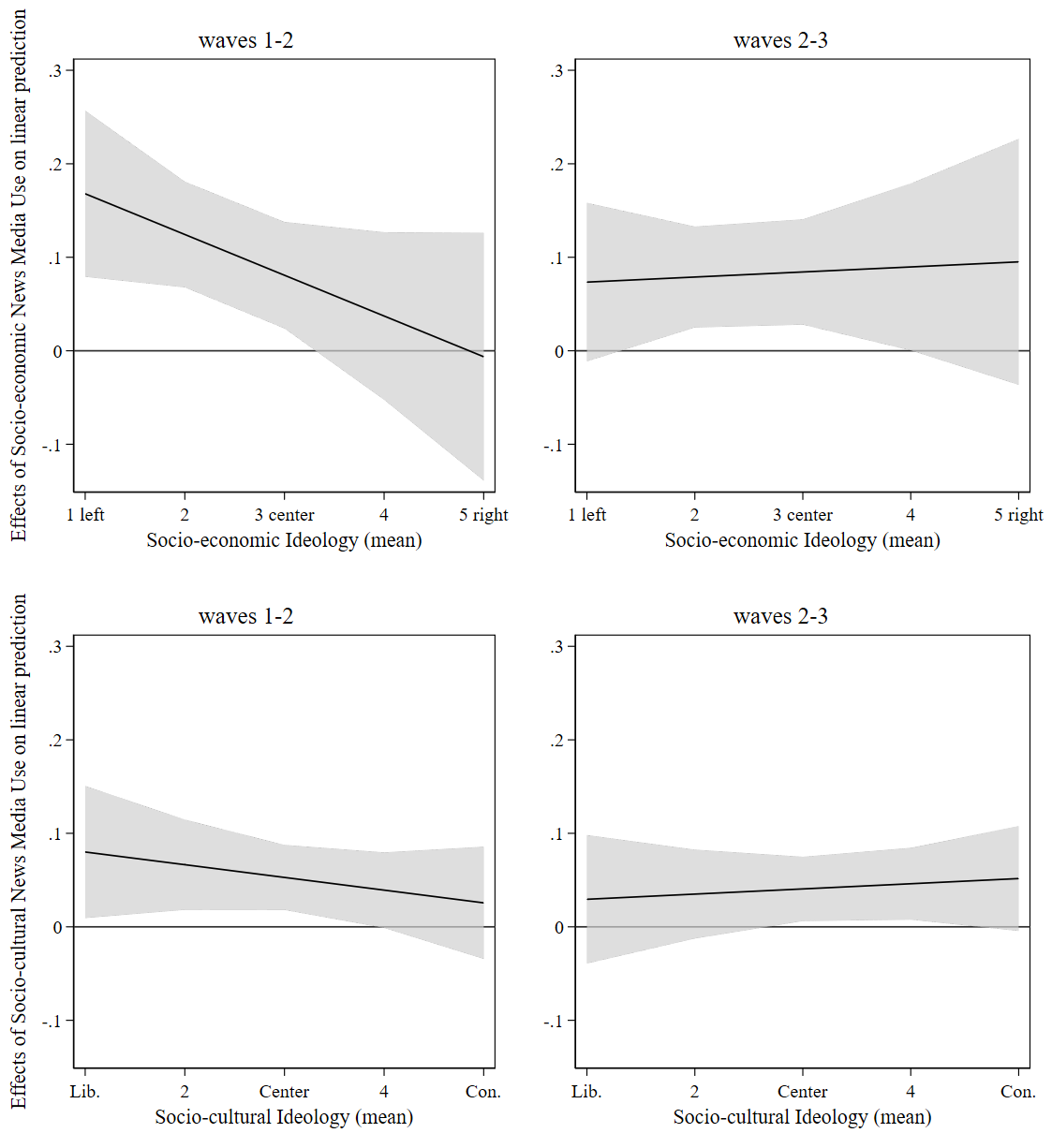


*Note:* Estimates in the models are under the control for sex, age, and education. Perceptions of violent crime are coded 1 = positive perceptions, 7 = negative perceptions. Both the ideological measures are coded 1-5, respectively: 1 = left, 3 = center, 5 = right, and 1 = culturally liberal, 3 = center, 5 = culturally conservative. n = 1 210.
